# Multitype drug interaction prediction based on the deep fusion of drug features and topological relationships

**DOI:** 10.1371/journal.pone.0273764

**Published:** 2022-08-29

**Authors:** Li-Ping Kang, Kai-Biao Lin, Ping Lu, Fan Yang, Jin-Po Chen

**Affiliations:** 1 School of Computer and Information Engineering, Xiamen University of Technology, Xiamen, China; 2 Engineering Research Center of Big Data Application in Private Health Medicine, Fujian Provincial University, Putian, China; 3 School of Economics and Management, Xiamen University of Technology, Xiamen, China; 4 Department of Automation, Xiamen University, Xiamen, China; Karunya Institute of Technology and Sciences, INDIA

## Abstract

Drug–drug interaction (DDI) prediction has received considerable attention from industry and academia. Most existing methods predict DDIs from drug attributes or relationships with neighbors, which does not guarantee that informative drug embeddings for prediction will be obtained. To address this limitation, we propose a multitype drug interaction prediction method based on the deep fusion of drug features and topological relationships, abbreviated DM-DDI. The proposed method adopts a deep fusion strategy to combine drug features and topologies to learn representative drug embeddings for DDI prediction. Specifically, a deep neural network model is first used on the drug feature matrix to extract feature information, while a graph convolutional network model is employed to capture structural information from the adjacency matrix. Then, we adopt delivery operations that allow the two models to exchange information between layers, as well as an attention mechanism for a weighted fusion of the two learned embeddings before the output layer. Finally, the unified drug embeddings for the downstream task are obtained. We conducted extensive experiments on real-world datasets, the experimental results demonstrated that DM-DDI achieved more accurate prediction results than state-of-the-art baselines. Furthermore, in two tasks that are more similar to real-world scenarios, DM-DDI outperformed other prediction methods for unknown drugs.

## 1. Introduction

When multiple drugs are taken together, unexpected drug–drug interactions (DDIs) may occur, which may have either beneficial or detrimental effects on treatment. Beneficial DDIs have a "1+1 > 2" synergistic effect and thus can be exploited as a safe and effective therapeutic strategy for severe diseases, such as cancer and AIDS [1]. In contrast, harmful DDIs may lead to serious adverse drug reactions and even threaten a patient’s life. Therefore, accurate identification of potential DDIs is an urgent and practical task. Currently, the main DDI identification methods need long-term clinical trials in vivo and in vitro, which are costly and time-consuming. If DDI can be effectively identified in advance using artificial intelligence (AI)-based computer approaches, the risk of medication coadministration will be reduced. Many methods have been proposed to predict DDIs, and these methods can be mainly divided into three categories.

Literature-based methods [2, 3] usually utilize natural language processing (NLP) techniques to extract important relationship information between drugs from scientific literature, electronic medical records, etc. The extracted text information is used as feature information to predict potential DDIs. For example, Shen et al. [4] proposed the knowledge-oriented representation learning method KMR, which collected drug pharmacology, drug classes, and drug textual description feature to learn unified drug embeddings for DDI prediction. These methods achieved satisfactory performance, but there are still many challenges with DDI extraction, such as the flexibility of the language, which is often not standardized.

Feature similarity-based methods [5, 6] assume that drugs with similar features may have the same drug interactions. Therefore, in early studies, a variety of machine learning approaches were proposed to build prediction models based on the similarity of drug-related features, such as profile fingerprints [7], chemical structures [8], pharmacological phenotypes [9], and RNA [10] for link prediction. Later, some efforts have been made to improve model accuracy by incorporating multiple feature information [11–13]. For instance, Chen et al. [1] integrated three types of feature information to predict the combination efficacy of drugs using a least squares classifier based on Laplace constraints. Cheng et al. [14] proposed an HNAI method, which combined four drug feature similarities and adopted four classifiers to build a prediction model. Gottlieb et al. [12] presented an INDI computational framework, which calculated seven feature similarities and used a logistic regression model for classification. These methods reasonably achieve better results than an individual feature, but it is challenging to collect multiple complete feature datasets. In addition, these methods fail to consider the topological relationships between drugs.

Network structure-based methods [15, 16] aim to project the topological network between drugs into low-dimensional space, and the learned embedding is treated as the potential drug features. There are three common methods for constructing networks. Factorization methods [17, 18] decompose the known DDI matrix into several low-dimensional matrices and reconstruct them for prediction tasks. For example, Yu et al. [19] developed a DDINMF method based on semi-nonnegative matrix factorization. Random walk methods [20, 21] perform random wander in the network and obtain sequences of nodes that can preserve the original topological information of the network. For instance, Park et al. [22] applied a random walk with a restart algorithm on the protein–protein network to identify DDIs. The neural network method [23, 24] leverages the powerful nonlinear learning ability of a neural network to obtain potential drug representations. For example, Zitnik et al. [16] constructed a multirelational network and used a graph convolutional network (GCN) as an encoder to obtain drug embeddings for DDI prediction. The above three types of methods consider the topological relationship between drugs and have attracted significant attention in bioinformatics network studies [15]. Subsequently, some methods for optimizing network topology information for specific DDI tasks have been developed [25–27]. For example, SkipGNN [25] aggregates the neighbor information within two hops for DDI prediction.

The latest studies tend to incorporate multiple drug-related entities (e.g., targets, enzymes, and pathways.) to construct heterogeneous drug graphs and use knowledge graph-based methods to predict DDIs. Many excellent methods have been developed, such as KGNN [28] and KG2E-capsule [29] methods, which better capture structural and semantic information and achieve satisfactory results. Comparisons with knowledge graph-based methods are not included in this study, as DM-DDI and other comparative methods are performed on homogeneous drug graphs.

Reviewing previous prediction methods, few works have focused on the deep fusion of drug features and topological relations. In these papers, the two types of information are usually combined relatively simply [30, 31], as in GCNs, where matrices containing the network structure and feature attributes are constructed and jointly input, and these features are propagated along the network. However, when the number of layers becomes large, oversmoothing problems may be encountered. To address the above deficiencies, we propose a deep fusion strategy to exchange and fuse the information between drug features and topological networks. Specifically, we set up two channels to process drug features and topology separately (capturing drug features with AE and relational networks with GCN), and also designed delivery operations between each layer to exchange information. Before the output layer, we use an attention mechanism to fuse the information from the two channels and obtain the final drug embedding, which is used for drug interaction prediction.

In addition, most traditional methods formulate DDI prediction as a binary classification problem, using "0" or "1" to indicate the presence or absence of a reaction. These methods do not describe whether the reactions are beneficial or harmful and are unable to provide useful guidance for coadministration. Therefore, several methods that can predict DDI-related events were proposed later [32–34]. For instance, Deng et al. [34] proposed DDIMDL, in which three features (substructure, target, and enzyme) were calculated and fed into the constructed submodels for separate training. The results of the submodels were combined using a deep neural network. Finally, 65 types of predicted labels were used as outputs, and each type of label corresponded to rich interaction content with chemical and pharmacological descriptions. The specific prediction results promoted the understanding of the underlying mechanisms behind adverse drug reactions. Inspired by this idea, our proposed model also predicts multiple types of DDI events.

Our contributions are summarized as follows:

1. DM-DDI provides a novel deep fusion strategy to fuse drug features and topological relationships, which helps to obtain informative and predictive drug representations. The great performance in comparison with multiple state-of-the-art methods validates the advantages of DM-DDI.
2. We use an attention mechanism to achieve a weighted fusion of drug features and network topology. Moreover, the distribution of attention scores can provide interpretability for the drug prediction process.
3. DM-DDI can predict many types of DDI events and is not limited to binary prediction results. The more detailed DDI prediction results can give us more insight into the coadministration of different drugs.

## 2. Materials and methods

### 2.1 Dataset

The DDI dataset used in the experiment comes from DDIMDL [34], which contains 37,264 pairwise interactions between 572 drugs characterized by four features: chemical substructure, target, enzyme, and pathway. Based on previous studies [34], we selected the three features with the best effects (chemical substructure, target, and enzyme) for similarity calculation.

We introduce how to calculate the similarity of drugs using the chemical structure as an example. PubChem defines 583 types of substructures (other feature descriptors are shown in Table 1), so each drug can be represented by a set of 583-bit feature descriptors with the value "1" or "0" indicating the presence or absence of the corresponding substructure. Finally, we can obtain a feature matrix of the chemical structures with the shape of (572, 583), and the same operation is performed on the target and enzyme features. Since the obtained feature matrices have high dimensionality and contain many "0" values, which may degrade the performance of the model, we conduct the Jaccard similarity calculation for each feature matrix to mitigate the impact, as shown in Eq (1).

$$J\left(di,dj\right)=\frac{\left|di\cap dj\right|}{\left|di\cup dj\right|}=\frac{\left|di\cap dj\right|}{\left|di\right|+\left|dj\right|-|di\cap dj|}$$ (1)

Where *di* and *dj* represent bit vectors drugs *i* and *j*, respectively; |*di* ∩ *dj|* is the intersection of *di* and *dj*, and |*di* ∩ *dj|* is the union. After the Jaccard similarity calculation, we can obtain three 572 × 572 feature similarity matrices (*X*_*s*_, *X*_*t*_, *X*_*e*_), which are concatenated as an initialized feature matrix *X*. The feature vector of drug *i* is represented as shown in Eq (2), and the symbol ⊕ denotes the concatenation operation.


$$X_{di}=X_{s}^{i}⊕X_{t}^{i}⊕X_{e}^{i}$$ (2)

**Table 1:** DDIMDL dataset.

#Drugs	#Interactions	Drug features			
		#Substructure	#Target	#Enzyme	#Pathway
572	37,264	583	1,162	202	957

### 2.2 Overview

**Fig 1:**
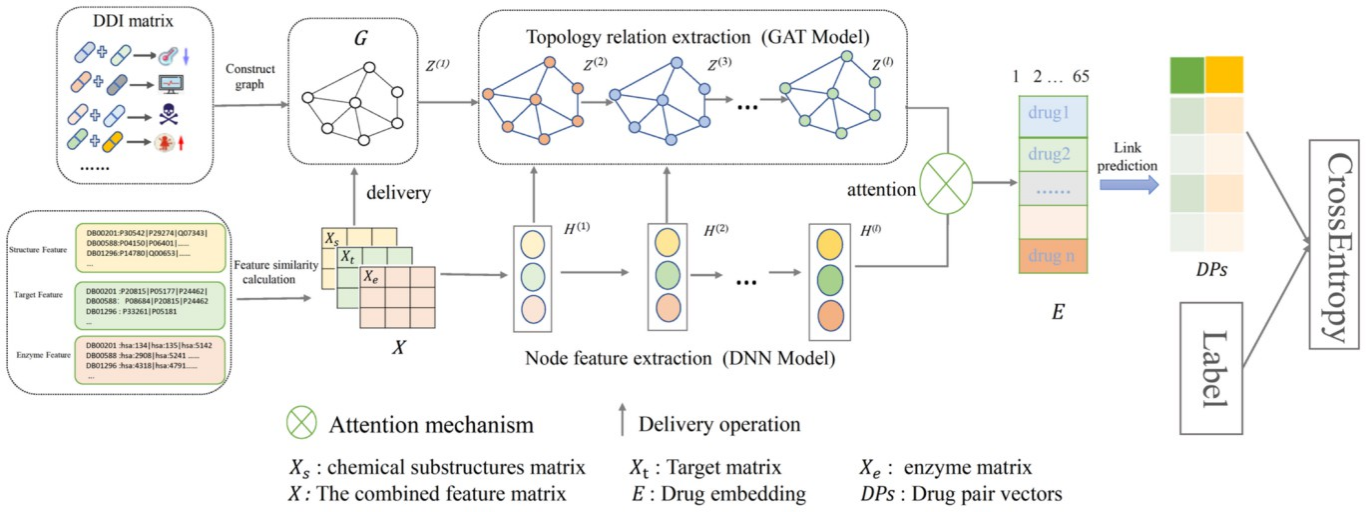
Overall framework of the proposed model. The changes in the node colors indicate the process of node learning, and *H*^(*l*)^ and *Z*^(*l*)^ indicate the vector representations learned at the *l*_*th*_ layers of the DNN model and GCN model, respectively.

The overall framework of DM-DDI is shown in Fig 1. The DDI matrix can be constructed as a network *G* = {*V*, *E*}, where the vertices *V* denote the drugs involved and the edges *E* denote the types of drug reactions. The three drug features (chemical substructure, target, and enzyme) can be calculated by Eq (1) to obtain three similarity matrices (*X*_*s*_, *X*_*t*_, *X*_*e*_), which are concatenated as a unified drug feature matrix *X*. The model is composed of four main modules: the node feature extraction module uses a DNN model on the feature matrix to capture drug feature information, and the learned feature embedding is represented by *H*; the structural relationship extraction module employs the GCN model on the constructed drug network to learn the structural information, and the learned structure embedding is represented by *Z*; the deep fusion module includes delivery operations between layers and an attention mechanism before the output layer, and the final representation *E* for all drugs can be obtained after deep fusion; and model optimization module adopts four different combination methods (Average, Hadamard, L1-norm, and Concatenation) to construct drug pair vectors (*DPs*) and uses the reaction types between the drug pairs as labels. Finally, the *DPs* and label are input into the cross-entropy loss function for optimization.

### 2.3 Node feature extraction module

To learn feature embeddings from raw data, we use the widely used autoencoder (AE). Because the AE model not only captures the nonlinear relationship between input and output quickly but also effectively reduces dimensionality. The AE model includes an encoder-decoder, where the encoder is acted by the DNN model. We treat the embedding obtained after encoding using Eq (3) as drug features. The details are as follows.

Assuming that the DNN model has *l* layers and that *H*^(*l*)^ denotes the embedding learned at layer *l*, we formulate *H*^(*l*)^ as follows.

$$H^{\left(l\right)}=\emptyset (W_{e}^{\left(l\right)}H^{\left(l-1\right)}+b_{e}^{\left(l\right)})$$ (3)

where ∅ represents the ReLU activation function, and *W*^(*l*)^ and *b*^(*l*)^ are the weight matrix and bias matrix at layer *l*_*th*_, respectively. *H*^(o)^ denotes the original feature matrix *X*. The decoder uses the inner product operation for decoding, and we train the model by minimizing the reconstruction loss.

### 2.4 Topology relation extraction module

The AE model can learn important features of each layer, e.g., *H*^(1)^, *H*^(2)^ ……*H*^(*i*)^, but it ignores the structural relationships between drugs. Therefore, we adopt the GCN model to extract the topological information between drugs. The structural embedding vector of layer *l*_*th*_ can be obtained from Eq (4).

$$Z^{\left(l\right)}=\emptyset ({\tilde{D}}^{-\frac{1}{2}}\tilde{A}{\tilde{D}}^{-\frac{1}{2}}Z^{\left(l-1\right)}W^{\left(l-1\right)})$$ (4)

where $\tilde{A}=A+I$ and $\tilde{D}=\sum_{j}{\tilde{A}}_{ij}.I$ is the identity diagonal matrix of the adjacent matrix $A.\tilde{A}$ is a self-loop matrix, and *$\tilde{D}$* is the degree matrix. *Z*^(*l*−1)^ is the embedding vector learned at layer (*l*0 − 1)_*th*_, and *W*^(*l*−1)^ is a trainable weight matrix that is used to map the information learned from layer (*l* − 1)_*th*_ to layer *l*_*th*_.

Considering that the AE model can obtain different levels of feature information, we integrate two embeddings between each layer to obtain a more informative drug representation, as shown in Eq (5).

$${\tilde{Z}}^{\left(l-1\right)}=\left(1-\alpha \right)Z^{\left(l-1\right)}+\alpha H^{\left(l-1\right)}$$ (5)

Where *α* is the fusion coefficient, which is used to control the fusion weight of the learned vectors between the GCN model and the AE model. Then, ${\tilde{Z}}^{\left(l-1\right)}$ is fed into the next GCN model to obtain the fused representation *Z*^(*l*)^, as shown in Eq (6).

$$Z^{\left(l\right)}=\emptyset ({\tilde{D}}^{-\frac{1}{2}}\tilde{A}{\tilde{D}}^{-\frac{1}{2}}{\tilde{Z}}^{\left(l-1\right)}W^{\left(l-1\right)})$$ (6)

where ∅ represents the ReLU activation function. In this way, the feature embedding *H*^(*l*−1)^ at layer (*l*−1)_*th*_ can be propagated through the normalized adjacency matrix and the same is true for the other layers.

### 2.5 Deep fusion

The key to the model is learning informative and high-quality drug embeddings, which help to promote the model performance. The deep fusion strategy uses delivery operations to realize intralayer and interlayer fusion and utilizes an attention mechanism to complete the final fusion. Eventually, the obtained drug embeddings can simultaneously retain the node features and topological relationships of different layers.

#### 2.5.1 Delivery operation

*(1) Intralayer fusion*. Between each layer, the drug feature embedding learned by the AE model is transferred to the GCN model with a certain weight ***α*** (refer to Eq (5)). Thus, ${\tilde{Z}}^{\left(l-1\right)}$ can accommodate two different types of information, i.e., drug features and the interaction between drugs.

*(2) Interlayer fusion*. Generally, the shallow layer (near the input) extracts low-level features, which contain more detailed information but more noise, and the deep layer (near the output) tends to extract high-level features with stronger semantic information but poorer detail perception. Therefore, effectively fusing information from different layers and overcoming the oversmoothing problem that GCN models may suffer is crucial to improving the overall model performance.

To the best of our knowledge, oversmoothing is mainly due to the overemphasis on the relationship with neighbor nodes while ignoring the node features during aggregation [30]. Kipf et al. [30] proposed residual connectivity to transfer the node feature information learned in the upper layer of the model to the next convolutional layer. Since then, a series of improved models have been proposed [35, 36]. For example, He et al. [36] proposed a model called LightGCN, which considers the representation of different GCN layers. In other words, this model mitigates the oversmoothing problem by integrating the granularity information of different layers. Inspired by this idea, we design Eqs (5) and (6) to cope with this problem. Specifically, we deliver the node feature information extracted from layer (*l* − 1)_*th*_ of the AE model to the GCN model for weight fusion. Then, the learned vector is used as feature vectors in the next iteration of the convolutional network to achieve interlayer fusion. Thus, the oversmoothing problem can be relieved by amplifying the node feature information. Bo et al. [37] adopted similar delivery operations in exchanging feature and topological information and demonstrated that such operations not only integrate structural information but also solve the GCN model’s oversmoothing problem.

#### 2.5.2 Attention mechanism

Considering the different importance of the two learned embeddings for the prediction task, it is necessary to adopt an attention mechanism to assign learnable weights to fuse.

Given *Z*^(*l*)^ and *H*^(*l*^, which are obtained from the GCN model and AE model at the last layer *l*, respectively, the attention mechanism is calculated as shown in Eq (7).

$$\left(\alpha_{z},\alpha_{h}\right)=att(Z,H)$$ (7)

where *α*_*Z*_ and *α*_*h*_ denote the attention coefficients of embedding *Z* and *H*, respectively. The detailed calculation process is as follows. Take node *i* as an example. Node *i* in embedding vector *Z* can be represented as *Z*^*i*^. We first apply a nonlinear transformation and multiply by the shared attention vector *q* to obtain its attention value $w_{z}^{i}$, as shown in Eq (8).

$${w_{z}^{i}=q}^{T}∙tanh(w∙{{(Z}^{i})}^{T}+b)$$ (8)

where *w* is the weight matrix, *b* is a bias vector, and *q* is a shared attention vector. We can also obtain the attention value $w_{h}^{i}$ of the drug feature embedding. Then, we use the SoftMax function to regularize the attention value and obtain its attention coefficient, as shown in Eq (9).


$$\alpha_{Z}^{i}=softmax\left(w_{z}^{i}\right)=\frac{exp(w_{z}^{i})}{exp\left(w_{z}^{i}\right)+exp(w_{h}^{i})}$$ (9)

Similarly, *$\alpha_{h}^{i}=softmax\left(w_{h}^{i}\right)$*. For n nodes, the weight coefficients *α*_*z*_ = [*α*_*z*_] and *α*_*h*_ = [*α*_*h*_], which are denoted *α*_*z*_ = diag(*α*_*z*_) and *α*_*h*_ = diag(*α*_*h*_), respectively. Finally, the final drug embedding representations *E* are obtained as shown in Eq (10).


$$E=\alpha_{Z}∙Z+\alpha_{h}∙H$$ (10)

### 2.6 Model optimization

After deep fusion, a 572 × 65 embedding vector *E* of all drugs are obtained, where row *i* and row *j* represent two drug vectors, denoted Ф(*di*) and Ф(*dj*), respectively. We provide four different combinations of methods to construct them into drug pair vectors (*DPs*), as shown in Table 2.

**Table 2:** Four different combinations of drug pairs.

Combination method	Dimensionality	Description
Average	d	$\Phi (di,dj)=\frac{1}{2}[\Phi (di)+\Phi (dj)]$
Hadamard	d	Φ(*di*, *dj*) = Φ(*di*) ⨀ Φ(*dj*)
L1-norm	d	Φ(*di*, *dj*) = |Φ(*di*) ⨀ Φ(*dj*)|
Concatenation	2d	Φ(*di*, *dj*) = Φ(*di*) ⨀ Φ(*dj*)

The symbol ⨀ indicates the Hadamard operation, while the symbol ⨁ indicates the concatenation operation. Note that the dimensionality of *DPs* changes only when the concatenation method is selected.

Suppose that the training set is *L* and that the set of label categories is *c*. Given a drug pair *i*, *i* ∈ *L*, we can calculate the cross-entropy loss by using Eq (11).


$$L=\sum_{l\in L}\sum_{i=1}^{c}Y_{\;li}ln{\hat{Y}}_{\;li}$$ (11)

The true label *$y_{i}^{c},c\in C$* indicates that drug pair *i* belongs to class *c*. The predicted label ${\hat{y}}_{i}^{c}$ denotes the probability that the predicted result belongs to class *c*. The prediction result for n drug pairs is denoted $\hat{Y}=\left[{\hat{\text{y}}}_{i}^{\text{c}}\right]\in {\mathbb{R}}^{n\times C}$, and the true representation is $Y=\left[{\text{y}}_{i}{}^{\text{c}}\right]\in {\mathbb{R}}^{n\times C}$.

## 3. Experimental results and discussion

### 3.1 Baselines

There are two types of comparison methods: feature-based methods and structure-based methods. For the feature-based methods, only the feature matrix is input. We compare DM-DDI with popular multiple reaction prediction models (DeepDDI, DDIMDL) as well as classical classifiers (LR, RF, and DNN). For structure-based methods, only the adjacency matrix is input. Representative embedding learning methods (LINE, HOPE, Node2Vec, and SDNE), as well as DPDDI and SkipGNN, are selected. Note that for the representative method, we could reimplement BioNEV [15] to save the learned embedding for downstream experiments.

- Cascade drug features
  We represent drug‒drug pairs as feature vectors and use the interaction types as labels. Then, they are fed into the classifier for training and prediction. These methods are traditional supervised learning methods, which we instantiate with logistic regression (LR) [38] and random forest (RF) [39] classifiers.
- DNN
  Deep neural networks (DNNs) and Lee’s [33] ideas are similar. They both directly concatenate three feature similarity matrices and feed them to a DNN classifier. The difference is that the DNN in this paper does not use autoencoders to reduce the dimension but directly combines the feature matrix as the input features instead.
- DeepDDI
  DeepDDI [32] selects a chemical structure feature matrix, which is dimensioned to 100 by principal component analysis (PCA), as a drug feature. Then, it is fed into a deep learning model for DDI prediction. In this work, we changed the output of the original implementation from the 86th class to the 65th class.
- DDIMDL
  DDIMDL [34] calculates the similarity of three drug-related features (substructure, target, enzyme). When combined directly, the different features may suffer from interference with each other. Thus three drug-related feature matrices are separately fed into three constructed submodels for training. Then, the DNN model is built for drug reaction prediction.
- HOPE
  HOPE [40] is a factorization method in which the model uses generalized singular value decomposition to decompose the adjacency matrix to maintain higher-order proximity.
- Node2Vec
  The graph embedding learned by node2vec [41] can represent both homogeneous similarity (the more shared neighbors there are, the greater the similarity) and structural similarity (the more similar the role that is played, the greater the similarity). The model adopts a biased random walk strategy to obtain neighbor nodes, and the hyperparameters are set to *p* = *q* = 1; therefore, the model can contain two different similarities.
- LINE
  LINE [42] focuses on learning embeddings based on distributional similarity. First-order nearest neighbors and second-order nearest s are trained separately, and then the two vectors are combined by the inner product as the vector representation.
- SDNE
  SDNE [43] utilizes deep AE models to maintain first- and second-order network proximity. This model uses highly nonlinear functions to obtain embeddings that retain local and global structural information.
- DPDDI
  The DPDDI model [26] uses graph convolutional networks (GCNs) to extract low-dimensional structural embeddings from the drug relationship network and then predicts the DDI using deep neural network (DNN) models.
- SkipGNN
  SkipGNN [25] receives messages from two-hop neighbors and immediate neighbors in the interaction network; thus, it captures higher-order topology information for DDI prediction.

### 3.2 Metric evaluation

To comprehensively evaluate the performance of DM-DDI, we use the accuracy (ACC), area under the precision-recall curve (AUPR), area under the receiver operating characteristic (ROC) curve (AUC), F1, precision (Pre), and recall as the evaluation metrics. We use micro metrics for AUPR and AUC and macro metrics for the other metrics (Pre_macro, F1_macro, Recall_macro). Pre_micro, Recall_micro, and F1_micro scores are the same as ACC scores in the multiclass task. Due to the imbalanced distribution of the DDI dataset, we focus on the AUPR_micro score, which provides a more accurate assessment of the model’s performance.

### 3.3 Experimental setup

The DM-DDI model utilizes multi-layer fusion with different numbers of nodes at different layers. We first discuss the effect of the number of layers on the experiment. In this paper, we consider five layers, and the number of nodes per layer is empirically set to {512, 1024, 2000, 256, 65}. We fix the number of neurons in the last hidden layer to 65, as there are 65 types of DDI events. Then, the number of layers is gradually increased to see how the number of layers affects the experimental results. The model learning rate hyperparameter is set to 0.003, and the number of epochs is set to 1,000, as the loss curve does not change when the number of epochs is close to 1,000. We apply fivefold cross-validation and randomly split all DDI pairs into five subsets in our experiments. We train models based on DDIs in the training set and then make predictions for DDIs in the test set. The evaluation score is the average of the output of the five rounds. We select the Adam optimizer to optimize DM-DDI and use the early-stopping strategy to prevent overfitting, which automatically stops the training if no improvement is observed after 20 epochs. The parameters of the other comparative models follow the original paper.

### 3.4 Experimental results

#### 3.4.1 Comparison with state-of-the-art methods

To make a fair comparison, we uniformly saved the results learned by the model as embedding vectors and then classified them with a DNN classifier. All results are evaluated by 10 runs of validations under the same conditions. The results are shown in Table 3, and the best results are marked in bold.

**Table 3:** Performance of our model against competitive approaches.

Method	ACC	AUPR_micro	AUC_micro	F1_macro	Pre_macro	Recall_macro
LR	0.721	0.785	0.993	0.306	0.504	0.254
RF	0.772	0.846	0.995	0.481	0.713	0.408
DNN	0.880	0.913	0.996	0.722	0.805	0.703
DeepDDI	0.837	0.890	0.996	0.685	0.728	0.661
DDIMDL	0.885	0.921	0.998	0.759	0.847	0.718
LINE	0.883	0.949	0.999	0.750	0.774	0.746
HOPE	0.901	0.952	0.999	0.762	0.794	0.749
Node2Vec	0.903	0.962	0.999	0.780	0.806	0.770
SDNE	0.777	0.848	0.995	0.466	0.579	0.441
DPDDI	0.784	0.860	0.996	0.491	0.587	0.454
SkipGNN	0.758	0.863	0.855	0.755	0.773	0.759
DM-DDI	**0.908**	**0.964**	**0.999**	**0.852**	**0.879**	**0.839**

Table 3 shows that the DM-DDI model outperforms the other comparison methods, especially in the F1_macro, Pre_macro, and Recall_macro metrics (the highest F1_macro in the comparison method is 0.780, while the score of F1_macro in DM-DDI model is 0.852). The result demonstrates the great performance of the DM-DDI, which may benefit from incorporating different levels of feature and structural information. An interesting phenomenon is observed: the Node2Vec model works best among the structure-based methods. A possible reason is that it incorporates two types of similarities (homogeneous and structural similarities) and therefore can better represent the topological relationships of neighbors. In contrast, the DPDDI and SDNE models seem to underperform, which we speculate may be caused by data imbalance. Because the imbalanced dataset has a great impact on the GCN convolution effect [44], the encoders of the DPDDI and SDNE models exactly use the GCN model. Note that the DM-DDI model also captures topology information by using the GCN module.

#### 3.4.2 Ablation study

To explore the effectiveness of each model component, we set the following ablation variants (shown in Table 4) for ablation experiments.

- **DM-DDI**_**w/o**_ **AE**: This variant has no AE module and sets the fusion coefficient alpha = 0. The DM-DDI model degrades to a multilayer GCN model; thus, the model captures topological information.
- **DM-DDI**_**w/o**_ **GCN**: This variant has no GCN module, and the embedded representation obtained by the AE model is directly used for end-to-end training. Only the attribute feature information of the drugs is used.
- **DM-DDI**_**w/o**_ **Att**: This variant has no attention mechanism. The obtained embeddings after GCN fusion are summed with the embedding learned by AE as the final drug embedding vector. This ablation variant is used to explore the impact of the attention mechanism.
- **DM-DDI**_**w/o**_ **Delivery**: This variant has no delivery operation for cross-fusion. Two embeddings from the GCN and AE modules are input into the attention mechanism to obtain the fused embedding vector. This variant is designed to verify the importance of the delivery operation.

**Table 4:** Ablation variants settings.

	_AE	_GCN	_Delivery	_Att
DM-DDI w/o AE		✓		
DM-DDI w/o GCN	✓			
DM-DDI w/o Att	✓	✓	✓	
DM-DDI w/o Delivery	✓	✓		✓

**Fig 2:**
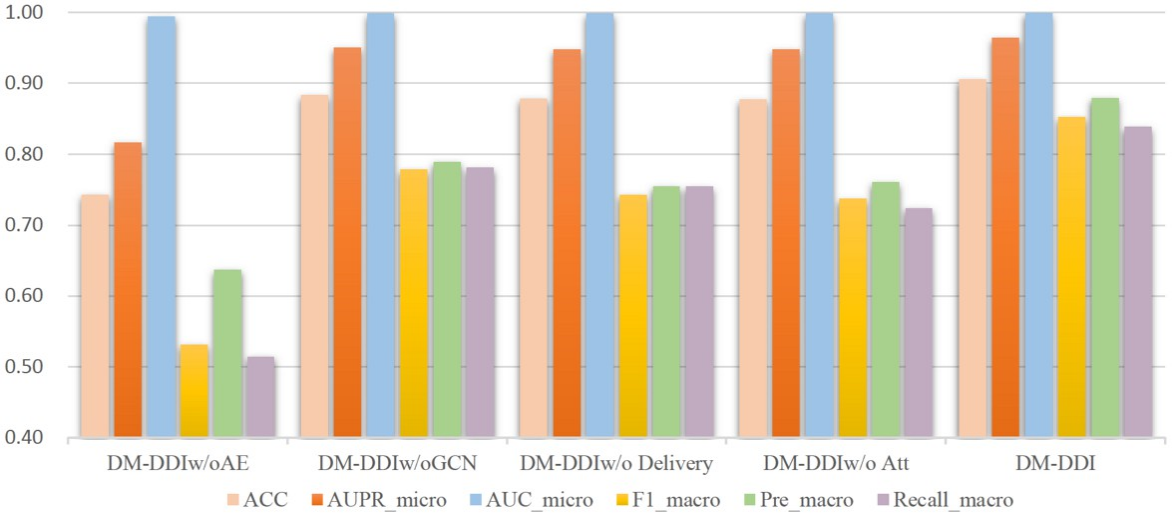
Comparison of the ablation experiment results.

The results from the bar chart in Fig 2 show that DM-DDI exceeds all ablation variants in all metrics, demonstrating the validity of each module of the model. Specifically, the ablation variant DM-DDI_w/o_ AE significantly decreases in numerous metrics compared to the DM-DDI model, indicating that drug features contribute significantly to the prediction of multiple drug reactions.

#### 3.4.3 Multi-class DDI dataset for link prediction

**Fig 3:**
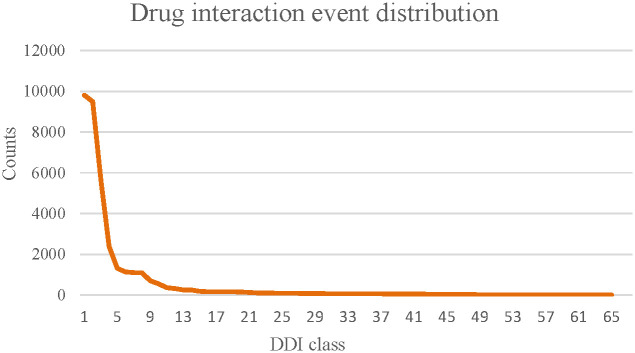
Statistical analysis of the DDI class distribution.

To verify the speculation in Section 3.4.1, we counted the reaction events for all classes and the results are shown in Fig 3. The results show that the distribution is unbalanced over 65 classes: the number of DDI events decreases significantly as the number of classes increases, with only a few numbers in the final class. To explore more deeply, we remove classes with few labels at the end and extract the first 30 classes of the DDI dataset (termed Class_30), and also select the first 10 classes of the DDI dataset (termed Class_10) with the popular multitype DDI prediction models (DeepDDI, DPDDI, and DDIMDL) for link prediction experiments. The experimental results are shown in Table 5.

**Table 5:** Comparison of the link prediction results obtained over multiple classes.

	Method	ACC	AUPR_micro	AUC_micro	F1_macro	Pre_macro	Recall_macro
Class_65	DeepDDI	0.837	0.890	0.996	0.685	0.728	0.661
	DPDDI	0.784	0.860	0.996	0.491	0.587	0.454
	DDIMDL	0.885	0.921	0.998	0.759	0.847	0.718
	DM-DDI	**0.906**	**0.964**	**0.999**	**0.852**	**0.879**	**0.839**
Class_30	DeepDDI	0.825	0.901	0.994	0.758	0.785	0.738
	DPDDI	0.789	0.867	0.992	0.610	0.657	0.583
	DDIMDL	0.889	0.939	0.996	0.856	0.888	0.835
	DM-DDI	**0.912**	**0.970**	**0.999**	**0.910**	**0.908**	**0.916**
Class_10	DeepDDI	0.845	0.920	0.988	0.819	0.830	0.808
	DPDDI	0.812	0.890	0.983	0.747	0.803	0.713
	DDIMDL	0.897	0.949	0.992	0.876	0.891	0.863
	DM-DDI	**0.923**	**0.978**	**0.999**	**0.926**	**0.912**	**0.923**

**Fig 4:**
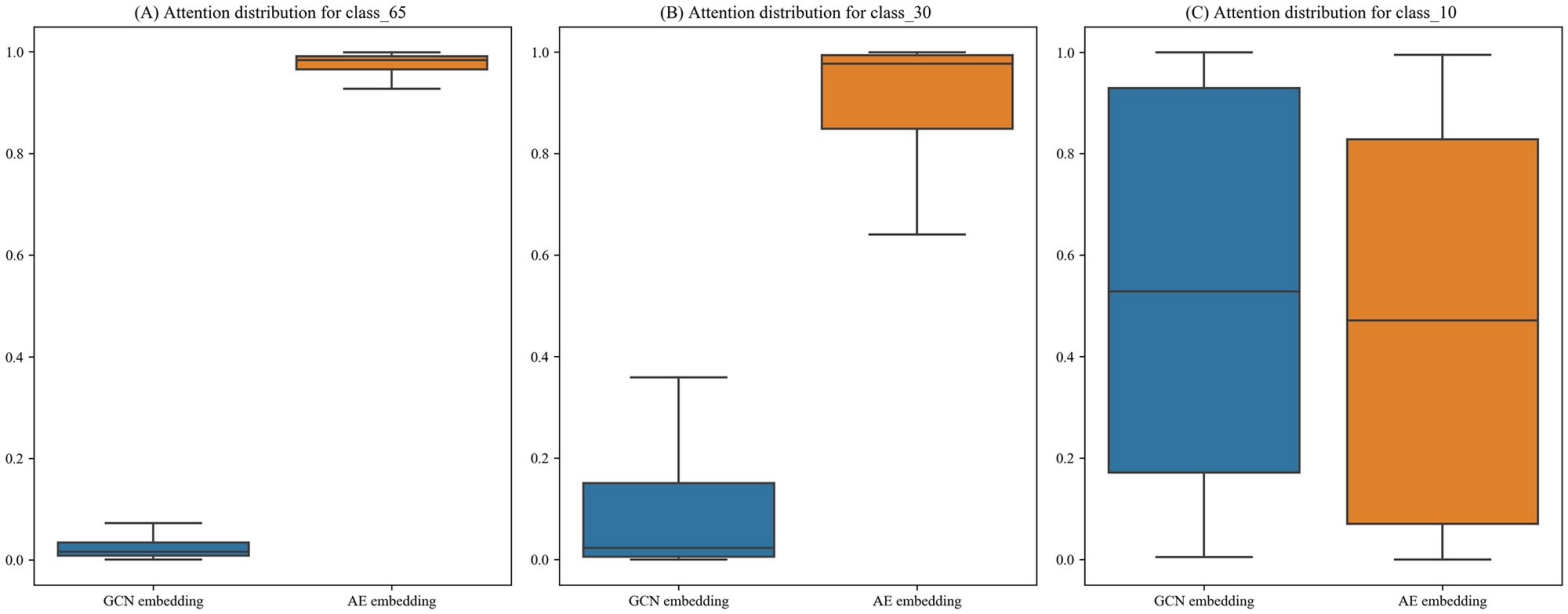
Distributions of the attention values over different classes. (A) Attention distribution for Class_65. (B) Attention distribution for Class_30. (C) Attention distribution for Class_10.

As seen from the experimental results in Table 5, when there are fewer classes, all models obtain higher scores on multiple metrics. This demonstrates that the performance of the models is affected by the data balance. The proposed model exhibits optimal performance in all cases, which shows that DM-DDI has a stable network structure. In addition, DPDDI experiences the most significant increase, with the value of F1_macro increasing by 25.6% from 0.491 in the Class_65 dataset to 0.747 in the Class_10 dataset. The observed increase could be attributed to a better convolution effect of the GCN encoder in DPDDI. Moreover, we output the distribution of attention values on different classes as auxiliary information. The results are shown in Fig 4. As the DDI dataset class decreases, the weight of attention values for the GCN embedding continues to increase and even exceeds that of the AE. This suggests that the ability of the GCN model to capture network topology information increases, leading to a higher contribution to the DDI prediction. These two findings further support the experimental speculation in Section 3.4.1. According to the above observations, we can infer that the GCN component can work better when the classes become more balanced and DM-DDI might achieve better results.

#### 3.4.4 Multi-task analysis

**Fig 5:**
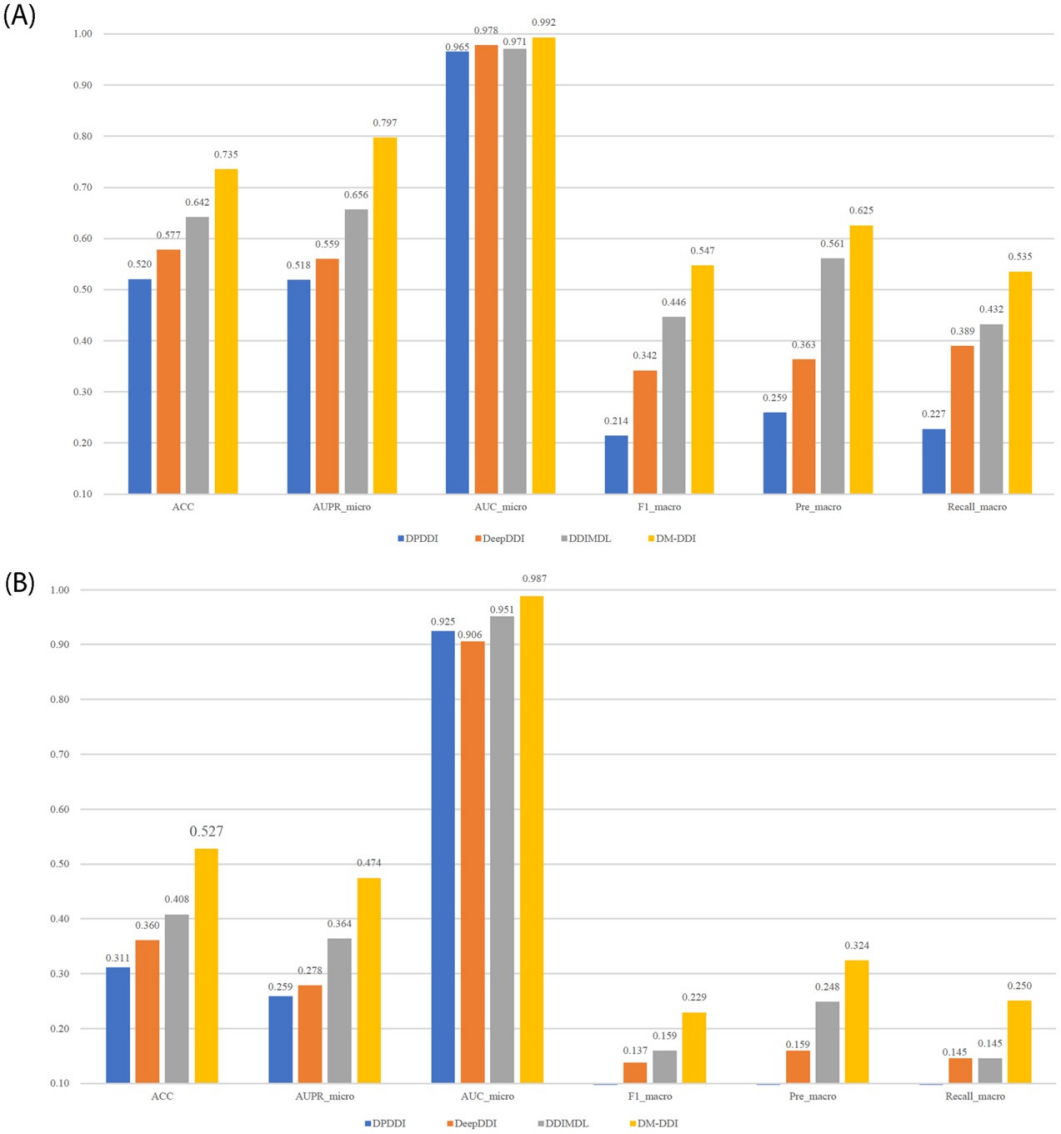
Experimental results in different tasks. (A) Task A. (B) Task B.

Usually, we are more concerned with the ability to predict unknown drugs, so we design tasks A and B that are closer to real cases for the experiment. Task A aims to predict the reaction between known drugs and unknown drugs, and Task B predicts the reaction between two new drugs. The two tasks differ from previous experiments, which predict unobserved reactions among known drugs, while this section predicts reactions with new drugs. Another difference is that the dataset in this section is divided based on drugs rather than drug pairs, and we randomly divide 572 drugs into 5 subsets and take 20% (115 drugs) for testing to simulate drugs without known interactions. In Task A, DM-DDI is performed on the training drugs and tested between the training drugs and testing drugs. In Task B, model training is also performed on the training drugs, whereas the predictions are all taken on the testing drugs, which is equal to predicting two completely new drugs. The experimental results are shown in Fig 5.

The results from the two tasks show that the performance of all models drops obviously when predicting new drugs. However, the proposed model significantly outperforms other competitive models in all metrics. For example, in Fig 5B, the DM-DDI model outperforms the second-best DDIMDL model in six metrics, namely, ACC, AUPR_micro, AUC_micro, F1_macro, Pre_macro, and Recall_macro, by 11.9%, 11.1%, and 3.6%, 7%, 7.6%, and 10.5%, respectively, which demonstrates the effectiveness of the model for new drug predictions. The advantage of prediction may benefit from the mutual reinforcement of drug features and topological information, and DM-DDI can make predictions from drug features even without neighbors.

#### 3.4.5 Sensitivity analysis

**Fig 6:**
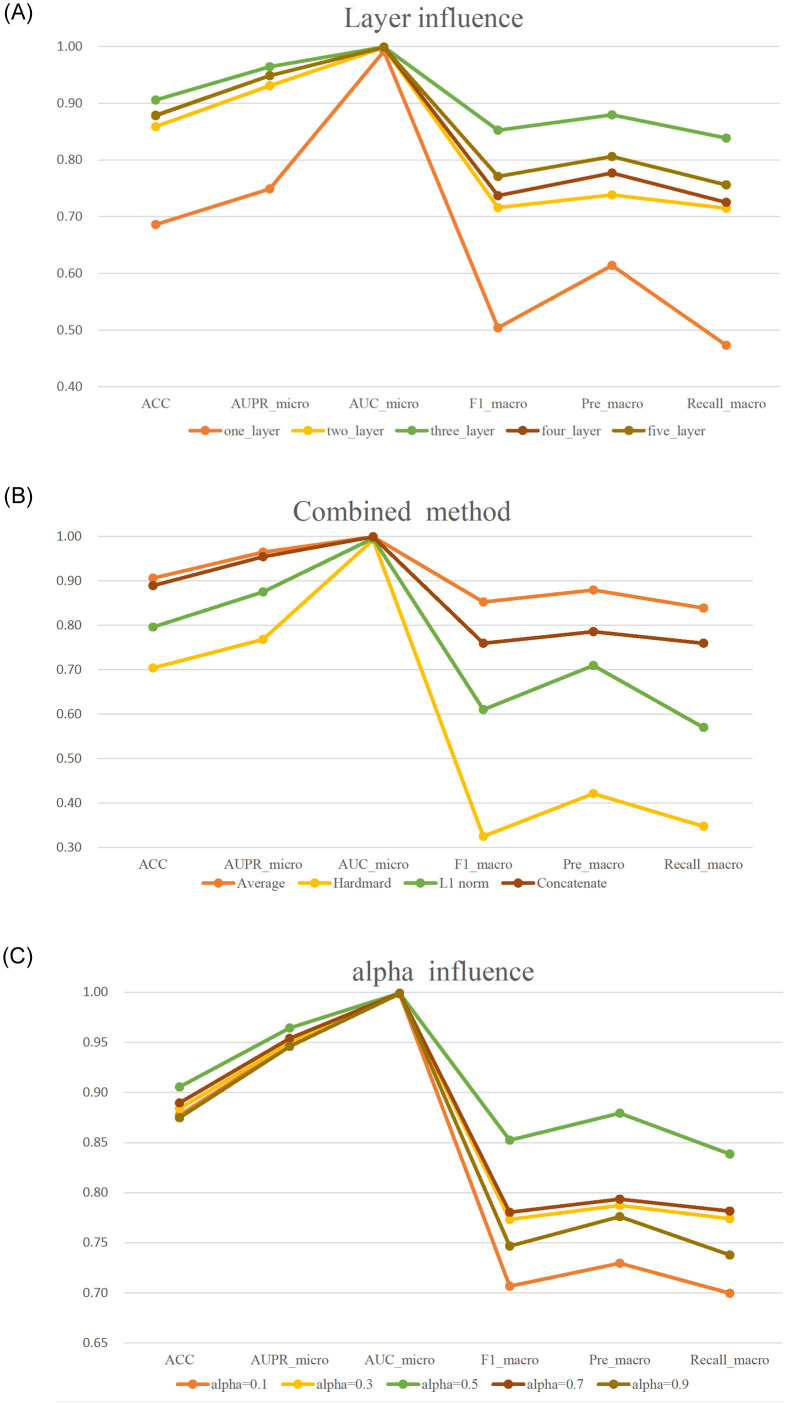
Results of ablation experiments. (A) Effect of the number of GCN layers. (B) Effects of drug combination methods. (C) Effect of the fusion coefficient.

In our work, three essential parameters are included: the coefficient 𝛼, the number of fusion layers, and the drug pair combination method. The set of *α* is given as {0.1, 0.3, 0.5, 0.7, 0.9}, the number of fusion layers varies from 1 to 5 layers, and the combination methods include the Average, Hadamard, L1-norm, and Concatenation methods. We conduct experiments to analyze the influences of parameters according to the prediction performances and fix the specific parameter with the best results. The experimental results are given in Fig 6.

As shown in Fig 6A, when the number of GCN layers is 3, the model achieves the best results. This outcome may be attributed to the appropriate capture of topological relations and drug feature information. Too few layers cannot capture sufficient information, while too many layers introduce noise and degrade the model performance. Therefore, we fix the number of layers to 3, and the number of hidden layer nodes is set to {2000, 256, 65}. In Fig 6B, we can see that when utilizing the average method, the model performs best overall, which means that the average combination approach can maintain the meanings of drug pairs well. The results in Fig 6C show that the best result is obtained when the fusion coefficient *α* is set to 0.5, namely, when the GCN embedding is equally fused with the AE embedding.

#### 3.4.6 Case study

A desirable DDI prediction model should not only pursue good prediction accuracy but also pursue the ability to accurately predict the types of drug interactions [45]. Therefore, this experiment conducts 65 types of DDI event prediction among 572 drugs and 37,264 drug pairs with known interactions. We predict the remaining 289,920 unknown drug reactions to verify the model’s ability. We focus on the top 10 classes of DDI events with the highest frequencies and output DDI events with the highest prediction scores. We find evidence in DrugBank [46] for verification, and the experimental results are shown in Table 6. Out of the top ten predicted DDI events with the highest scores, eight reactions are confirmed. For example, the true drug interaction type that occurs between abiraterone and fentanyl is “1”, and the predicted probability that falls into type “1” is 0.93. Therefore, the prediction is correct, which means that the metabolism of fentanyl can be decreased when combined with abiraterone.

**Table 6:** Case study prediction results.

Drug1	Drug2	Score	Label	Evidence	Description
Abiraterone	Fentanyl	0.943	1	Drugbank	The metabolism of fentanyl can be decreased when combined with abiraterone.
Acetylcholine	Cinchocaine	0.970	2	Drugbank	The risk or severity of adverse effects can be increased when cinchocaine is combined with acetylcholine.
Naproxen	Clofarabine	0.949	3	Drugbank	Clofarabine may decrease the excretion rate of naloxone which could result in a higher serum level.
Apalutamide	Betrixaban	0.939	4	Drugbank	The serum concentration of betrixaban can be decreased when it is combined with apalutamide.
Acetohexamide	Memantine	0.969	5	Drugbank	The excretion of memantine can be decreased when combined with acetazolamide.
Hydroxyzine	Cisplatin	0.975	6	N.A.	The metabolism of hydrocodone can be decreased when combined with cisplatin.
Bortezomib	Hydroxyzine	0.889	7	Drugbank	The risk or severity of QTc prolongation can be increased when bortezomib is combined with hydroxyzine.
Alimemazine	Eprosartan	0.872	8	Drugbank	Eprosartan may increase the hypotensive activities of amifostine.
Amobarbital	Fenofibrate	0.834	9	Drugbank	The metabolism of fenofibrate can be increased when combined with amobarbital.
Droxidopa	Loxoprofen	0.920	10	N.A.	The risk or severity of hypertension can be increased when droxidopa is combined with loxoprofen.

N.A.: evidence not available.

**Fig 7:**
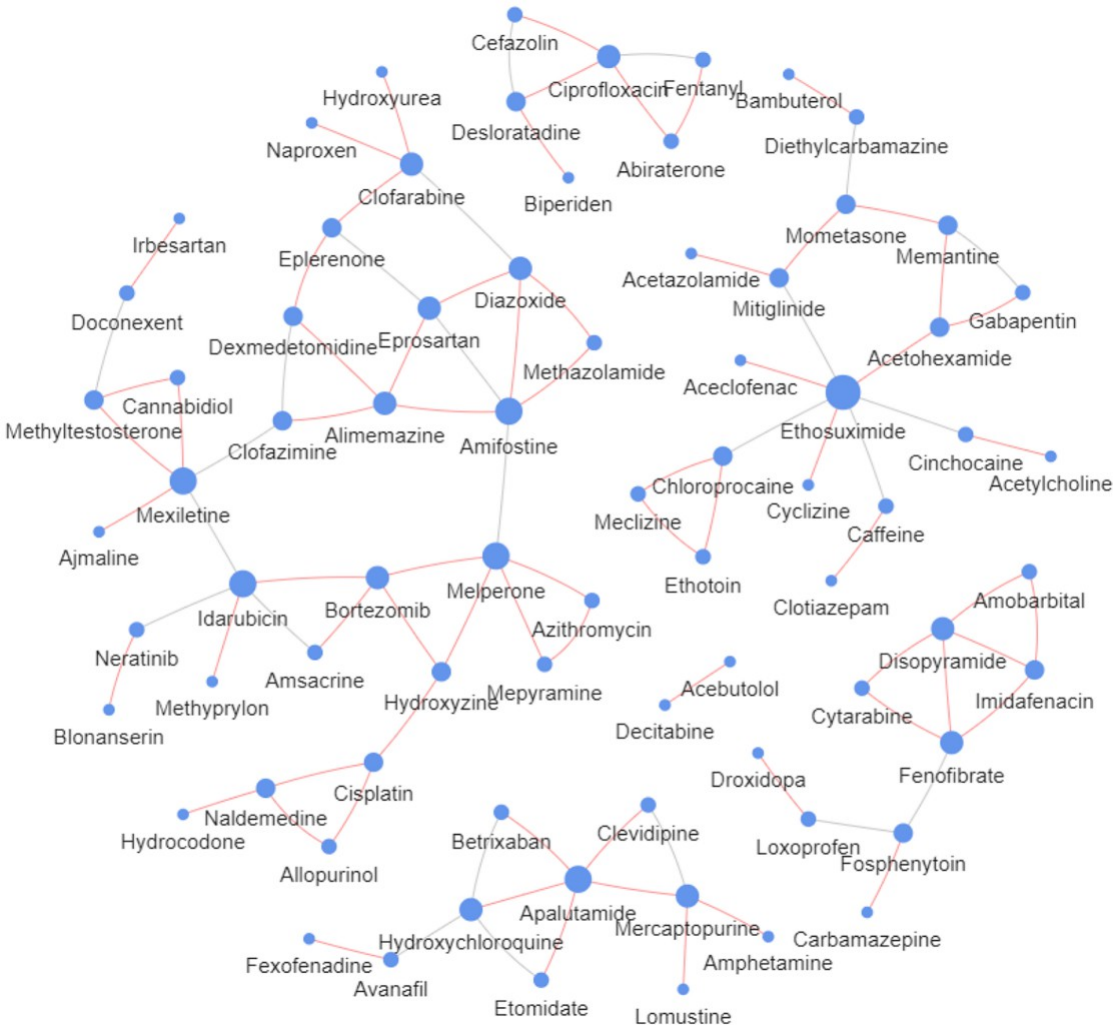
Top 100 pairs of DDI prediction results.

Furthermore, we counted the top 100 drug pairs with the highest prediction scores, and the results are displayed in Fig 7. The size of the drug node correlates with the number of reactions; the red edge indicates that the interaction is confirmed, and the grey edge indicates that no evidence is found. Sixty-nine percent of the reactions in the graph are validated. The case study results show that the proposed model can predict the unknown DDI events well.

## 4. Conclusion

We propose a novel drug interaction prediction model (DM-DDI), which deeply fuses drug features with topological information of neighbors through delivery operation and attention mechanisms. Finally, the information-rich drug embedding vectors are obtained by end-to-end training. The experimental results showed that DM-DDI outperformed numerous competitive methods, even on unbalanced 65 class DDI datasets. The prediction accuracy can reach 0.908, and the AUPR can reach 0.964. The case study’s prediction results are well-readable and more accurate, which provides strong support for drug interaction prediction. However, our model still has the following limitations: only a single drug–drug relationship was used to capture topological information. In the future, we will collect multitype drug-related entities (e.g., drugs, proteins, diseases, and side effects) and construct multi-relationship networks to characterize the structural embedding of drugs. In addition, we only utilized basic model components to learn feature representations, such as the GCN model used to capture topological information. Later, we can replace them with more advanced models to better address the imbalance problem.

## References

[1] Chen X, Ren B, Chen M, Wang Q, Zhang L, Yan G, et al. NLLSS: Predicting Synergistic Drug Combinations Based on Semi-supervised Learning. PLoS computational biology 2016;12(7):e1004975. 10.1371/journal.pcbi.1004975 27415801 PMC4945015

[2] Sun X, Dong K, Ma L, Sutcliffe R, He F, Chen S, et al. Drug-Drug Interaction Extraction via Recurrent Hybrid Convolutional Neural Networks with an Improved Focal Loss. Entropy. 2019;21(1). 10.3390/e21010037 33266753 PMC7514143

[3] Lim S, Lee K, Kang J. Drug drug interaction extraction from the literature using a recursive neural network. PLoS One. 2018;13(1):e0190926. 10.1371/journal.pone.0190926 29373599 PMC5786304

[4] Shen Y, Yuan K, Yang M, Tang B, Li Y, Du N, et al. KMR: knowledge-oriented medicine representation learning for drug-drug interaction and similarity computation. J Cheminformatics. 2019:11(1): 1–6. Epub 2019/03/16. 10.1186/s13321-019-0342-y 30874969 PMC6419809

[5] Yan C, Duan G, Zhang Y, Wu F-X, Pan Y, Wang J, editors. IDNDDI: An Integrated Drug Similarity Network Method for Predicting Drug-Drug Interactions. International Symposium on Bioinformatics Research and Applications; 2019: Springer. 10.1007/978-3-030-20242-2_8

[6] Chen X, Zhou C, Wang C-C, Zhao Y. Predicting potential small molecule–miRNA associations based on bounded nuclear norm regularization. Briefings Bioinf. 2021;22(6):bbab328. 10.1093/bib/bbab328 34404088

[7] Vilar S, Uriarte E, Santana L, Tatonetti NP, Friedman C. Detection of drug-drug interactions by modeling interaction profile fingerprints. PLoS One. 2013;8(3):e58321. 10.1371/journal.pone.0058321 23520498 PMC3592896

[8] Takeda T, Hao M, Cheng T, Bryant SH, Wang YJJoc. Predicting drug–drug interactions through drug structural similarities and interaction networks incorporating pharmacokinetics and pharmacodynamics knowledge. J Cheminformatics. 2017;9(1):1–9. 10.1186/s13321-017-0200-8 28316654 PMC5340788

[9] Peng L, Chao H, Fu Y, Wang J, Wu Z, Ru J, et al. Large-scale exploration and analysis of drug combinations. Bioinformatics. 2015;(12):2007. 10.1093/bioinformatics/btv080 25667546

[10] Wang CC, Zhu CC, Chen XJBiB. Ensemble of kernel ridge regression-based small molecule–miRNA association prediction in human disease. Briefings Bioinf. 2022;23(1):bbab431. 10.1093/bib/bbab431 34676393

[11] Yan X, Yin P, Wu X, Han JJFip. Prediction of the Drug–Drug Interaction Types with the Unified Embedding Features from Drug Similarity Networks. Front Pharmacol. 2021;12. 10.3389/fphar.2021.794205 34987405 PMC8721167

[12] Gottlieb A, Stein GY, Oron Y, Ruppin E, Sharan R. INDI: a computational framework for inferring drug interactions and their associated recommendations. MOL SYST BIOL. 2012;8:592. Epub 2012/07/19. 10.1038/msb.2012.26 22806140 PMC3421442

[13] Andrej K, Polonca F, Brane L, Yang JMJPO. Predicting potential drug-drug interactions on topological and semantic similarity features using statistical learning. PLoS One. 2018;13(5):e0196865. 10.1371/journal.pone.0196865 29738537 PMC5940181

[14] Cheng F, Zhao ZJJotAMIAJ. Machine learning-based prediction of drug-drug interactions by integrating drug phenotypic, therapeutic, chemical, and genomic properties. JAMIA. 2014;21(e2):278–86. 10.1136/amiajnl-2013-002512 PMC4173180 24644270

[15] Yue X, Wang Z, Huang J, Parthasarathy S, Moosavinasab S, Huang Y, et al. Graph embedding on biomedical networks: methods, applications and evaluations. Bioinformatics. 2020;36(4):1241–51. 10.1093/bioinformatics/btz718 31584634 PMC7703771

[16] Zitnik M, Agrawal M, Leskovec JJB. Modeling polypharmacy side effects with graph convolutional networks. Bioinformatics. 2018;34(13):i457–i66. 10.1093/bioinformatics/bty294 29949996 PMC6022705

[17] Rohani N, Eslahchi C, Katanforoush A. Iscmf: Integrated similarity-constrained matrix factorization for drug–drug interaction prediction. Network Modeling Analysis in Health Informatics and Bioinformatic. 2020;9(1):1–8. 10.1007/s13721-019-0215-3

[18] Zhu J, Liu Y, Zhang Y, Li DJIJoB, Informatics H. An Attribute Supervised Probabilistic Dependent Matrix Tri-Factorization Model for the Prediction of Adverse Drug-Drug Interaction. IEEE J Biomed Health Inform. 2020;PP(99):1–11. 10.1109/jbhi.2020.3048059 33373310

[19] Yu H, Mao KT, Shi JY, Huang H, Chen Z, Dong K, et al. Predicting and understanding comprehensive drug-drug interactions via semi-nonnegative matrix factorization. BMC Syst Biol. 2018;12(Suppl 1):14. https://doi.org/0.1186/s12918-018-0532-7 29671393 10.1186/s12918-018-0532-7 PMC5907306

[20] Zhang P, Wang F, Hu J, Sorrentino R. Label propagation prediction of drug-drug interactions based on clinical side effects. SCI REP-UK. 2015;5(1):1–10. 10.1038/srep12339 26196247 PMC5387872

[21] Chen Z-H, You Z-H, Guo Z-H, Yi H-C, Luo G-X, Wang Y-B. Prediction of Drug–Target Interactions From Multi-Molecular Network Based on Deep Walk Embedding Model. Front Bioeng Biotechnol. 2020;8:338. 10.3389/fbioe.2020.00338 32582646 PMC7283956

[22] Park K, Kim D, Ha S, Lee DJPO. Predicting Pharmacodynamic Drug-Drug Interactions through Signaling Propagation Interference on Protein-Protein Interaction Networks. PLoS One. 2015;10(10):e0140816. 10.1371/journal.pone.0140816 26469276 PMC4607460

[23] Cao S, Lu W, Xu Q, editors. Deep neural networks for learning graph representations. Proceedings of the AAAI Conference on Artificial Intelligence; 2016. 10.1609/aaai.v30i1.10179.

[24] Liu S, Huang Z, Qiu Y, Chen Y, Zhang W, editors. Structural Network Embedding using Multi-modal Deep Auto-encoders for Predicting Drug-drug Interactions. 2019 IEEE International Conference on Bioinformatics and Biomedicine (BIBM); 2019. 10.1109/BIBM47256.2019.8983337.

[25] Huang K, Xiao C, Glass L, Zitnik M, Sun J. SkipGNN: Predicting Molecular Interactions with Skip-Graph Networks. SCI REP-UK. 2020:10(1): 1–6. 10.1038/s41598-020-77766-9 33273494 PMC7713130

[26] Feng Y-H, Zhang S-W, Shi J-YJBb. DPDDI: a deep predictor for drug-drug interactions. BMC bioinformatics. 2020;21(1):1–15. 10.1186/s12859-020-03724-x 32972364 PMC7513481

[27] Wang Y, Min Y, Chen X, Wu J, editors. Multi-view Graph Contrastive Representation Learning for Drug-Drug Interaction Prediction. WWW ’21: The Web Conference 2021; 2021. 10.1145/3442381.3449786

[28] Lin X, Quan Z, Wang ZJ, Ma T, Zeng X, editors. KGNN: Knowledge Graph Neural Network for Drug-Drug Interaction Prediction. Twenty-Ninth International Joint Conference on Artificial Intelligence and Seventeenth Pacific Rim International Conference on Artificial Intelligence; 2020. 10.24963/ijcai.2020/380

[29] Su X, You Z-H, Huang D-s, Wang L, Wong L, Ji B, et al., editors. Biomedical Knowledge Graph Embedding with Capsule Network for Multi-label Drug-Drug Interaction Prediction. IEEE Transactions on Knowledge Data Engineering; 2022. 10.1109/TKDE.2022.3154792.

[30] Kip F TN, Welling M, editors. Semi-Supervised Classification with Graph Convolutional Networks. International Conference on Learning Representations; 2017. 10.48550/arXiv.1609.02907.

[31] Kipf TN, Welling M, editors. Variational Graph Auto-Encoders. Conference on Neural Information Processing Systems; 2016. 10.48550/arXiv.1611.07308.

[32] Ryu JY, Kim HU, Sang YL, editors. Deep learning improves prediction of drug–drug and drug–food interactions. Proceedings of the National Academy of Sciences; 2018. 10.1073/pnas.1803294115 PMC5939113 29666228

[33] Lee G, Park C, Ahn J. Novel deep learning model for more accurate prediction of drug-drug interaction effects. BMC Bioinformatics. 2019;20(1). 10.1186/s12859-019-3013-0 31387547 PMC6685287

[34] Deng Y, Xu X, Qiu Y, Xia J, Zhang W, Liu SJB. A multimodal deep learning framework for predicting drug–drug interaction events. Bioinformatics. 2020;36(15):4316–22. 10.1093/bioinformatics/btaa501 32407508

[35] Wang X, Li Z, Jiang M, Wang S, Zhang S, Wei ZJJoci. Molecule property prediction based on spatial graph embedding. J Chem Inf Model. 2019;59(9):3817–28. 10.1021/acs.jcim.9b00410.s001 31438677

[36] He X, Deng K, Wang X, Li Y, Zhang Y, Wang M. Lightgcn: Simplifying and powering graph convolution network for recommendation. J Chem Inf Model. 2019: 59(9): 3817–28. 10.1145/3397271.340106331438677

[37] Bo D, Wang X, Shi C, Zhu M, Lu E, Cui P, editors. Structural deep clustering network. Proceedings of The Web Conference 2020; 2020. 10.1145/3366423.3380214

[38] DeStefano JJ, editor Logistic regression and the Boltzmann machine. 1990 IJCNN International Joint Conference on Neural Networks; 1990: IEEE. 10.1109/ijcnn.1990.137845

[39] Breiman LJML. Random forest. Mach Learn. 2001;45:5–32.

[40] Ou M, Cui P, Pei J, Zhang Z, Zhu W, editors. Asymmetric transitivity preserving graph embedding. Proceedings of the 22nd ACM SIGKDD international conference on Knowledge discovery and data mining; 2016. 10.1145/2939672.2939751

[41] Grover A, Leskovec J, editors. node2vec: Scalable Feature Learning for Networks. the 22nd ACM SIGKDD International Conference; 2016. 10.1145/2939672.2939754 PMC5108654 27853626

[42] Tang J, Qu M, Wang M, Zhang M, Yan J, Mei Q, editors. Line: Large-scale information network embedding. Proceedings of the 24th international conference on world wide web; 2015. 10.1145/2736277.2741093

[43] Wang D, Cui P, Zhu W, editors. Structural deep network embedding. Proceedings of the 22nd ACM SIGKDD international conference on Knowledge discovery and data mining; 2016. https://doi.org/0.1145/2939672.2939753

[44] Shi M, Tang Y, Zhu X, Wilson D, Liu J, editors. Multi-Class Imbalanced Graph Convolutional Network Learning. Twenty-Ninth International Joint Conference on Artificial Intelligence and Seventeenth Pacific Rim International Conference on Artificial Intelligence {IJCAI-PRICAI-20}; 2020. 10.24963/ijcai.2020/398

[45] Han K, Cao P, Wang Y, Xie F, Ma J, Yu M, et al. A Review of Approaches for Predicting Drug–Drug Interactions Based on Machine Learning. Front Pharmacol. 2022:12: 814858. 10.3389/fphar.2021.814858 35153767 PMC8835726

[46] Alberta TUo, Knox C, Wilson M. DrugBank versions. 5.1.9. https://go.drugbank.com/

